# Factors affecting immunogenicity of BCG in infants, a study in Malawi, The Gambia and the UK

**DOI:** 10.1186/1471-2334-14-184

**Published:** 2014-04-05

**Authors:** Yun-Gyoung Hur, Patricia Gorak-Stolinska, Maeve K Lalor, Hazzie Mvula, Sian Floyd, John Raynes, Anne Ben-Smith, Joseph R Fitchett, Katie L Flanagan, Sarah Burl, Martin O Ota, Amelia C Crampin, Steven G Smith, Hazel M Dockrell

**Affiliations:** 1 Department of Immunology and Infection, Faculty of Infectious and Tropical Diseases, London School of Hygiene & Tropical Medicine London WC1E 7HT, UK; 2 Karonga Prevention Study, PO Box 46, Chilumba, Karonga District, Malawi; 3 Department of Infectious Disease Epidemiology, Faculty of Epidemiology & Population Health, London School of Hygiene & Tropical Medicine, London WC1E 7HT, UK; 4 Medical Research Council Unit, PO Box 273, Fajara, The Gambia; 5 Department of Microbiology and Institute of Immunology and Immunological Diseases, Yonsei University College of Medicine, 134, Sinchondong, Seodaemun-gu, Seoul 120-752, South Korea; 6 Current address: Yonsei University College of Medicine, Seoul, South Korea; 7 Current address: Public Health England, London, UK; 8 Current address: Monash University, Prahran, Victoria, Australia; 9 Current address: Paediatric Infectious Diseases, Imperial College London, London, UK

**Keywords:** BCG vaccination, CRP, Cytokine, Infant immune response, *M. tuberculosis* PPD, Zinc

## Abstract

**Background:**

BCG immunogenicity in infants differs between populations and these differences have been attributed to various factors. In this study, the influence of geographical location, season of birth, timing of vaccination, micronutrient status (zinc) and inflammatory status (C-reactive protein, CRP) were assessed.

**Methods:**

Immunogenicity was assessed by cytokine signature in culture supernatants from diluted whole blood samples stimulated with *M. tuberculosis* PPD, using a multiplex bead assay. Results were correlated with the plasma zinc and CRP concentrations at the time of sampling, and with interview and household data. BCG vaccinated infants were recruited in Malawi, The Gambia and the UK.

**Results:**

In Malawi, infants vaccinated within the first week after birth showed lower production of most cytokines measured than those vaccinated later. The number of cytokines showing significant differences between Malawian and Gambian infants decreased after adjusting for season of birth. In Malawi, a proportion of infants had zinc deficiency and elevated plasma CRP (>10 mg/L), but neither zinc deficiency nor high CRP was associated with production of any of the cytokines measured.

**Conclusions:**

The cytokine/chemokine signatures observed in response to *M. tuberculosis* PPD in infants at 3 months post BCG vaccination were affected by geographical location, season of birth, and timing of vaccination but not associated with the concentration of plasma zinc or inflammatory status. These factors should be considered in future trials of new TB vaccines.

## Background

Tuberculosis (TB) is caused by *Mycobacterium tuberculosis* (*M. tb*) and is one of the major global health challenges with 8.6 million incident cases worldwide [1]. Despite successful global TB control efforts and decreasing TB incidence [1], the variable efficacy and immunogenicity of the BCG vaccine in different populations [2-4] highlights the ongoing need to develop new vaccines or delivery strategies. Better understanding of the factors leading to variations in immune responses to BCG and how different immune responses correlate with the efficacy of BCG, may help to evaluate the efficacy of new TB vaccines.

Micronutrient deficiencies may impact on this immune response. Zinc has been classified by WHO as a “problem” micronutrient which requires supplementary provision to breast-milk fed infants from about 6 months of age [5]. Zinc deficiency causes an imbalance in immune function by shifting a Th1 to a Th2 response, which results in cell-mediated immune dysfunction that may increase susceptibility to various pathogens [6]. There have been reports showing the beneficial effect of zinc supplementation on the incidence of infectious diseases [7,8]. The best available biomarker to assess population zinc status is serum or plasma zinc concentration [9]. Zinc deficiency may occur as a result of inadequate dietary intake [10]. In Malawi, the most important food staple is maize and a maize porridge called nsima which has a high concentration of phytate. Phytate inhibits bioavailability of zinc [11] and may cause zinc deficiency, which may be exacerbated by limited animal protein intake in rural areas.

Inflammation, as a result of infection, may also influence the immune response following vaccination. C-reactive protein (CRP) is an acute phase protein which is released from the liver. It exists normally in trace levels in serum and rapidly increases in response to a variety of infections and inflammatory conditions [12]. Quantitative CRP concentration in plasma or serum has been used as a screening tool for bacterial and viral infection [13]; a range of 10–40 mg/L occurring in mild inflammation and viral infection, while a range of 40–200 mg/L is observed in active inflammation and bacterial infection [12]. CRP is also measured to help interpret the results of zinc assays as the concentration of serum zinc varies according to inflammation status [9].

Previous immunology studies undertaken at the Karonga Prevention Study (KPS) to compare the immune responses between Malawi and the UK, following BCG vaccination, showed different IFN-γ responses to mycobacterial antigens in adolescents and infants in these two populations. This was regardless of the extent of exposure to environmental mycobacteria, which has been the dominant hypothesis for interpreting variability in BCG vaccine-induced protection [4,14,15]. These results led us to investigate other potential factors which may influence infant immune responses to mycobacterial antigens. In this study, we looked at the cytokine/chemokine signatures in infants at 3 months post BCG vaccination. To assess the effects of geographical location and the environment, infants born in different seasons in Malawi and The Gambia were examined. The latitude of the study sites, Karonga in Malawi, Sukuta in The Gambia and London in the UK are 10°S, 13°N and 51°N respectively, and it was hypothesized that the geographical similarities between Malawi and The Gambia might lead to cytokine/chemokine signatures that would be more similar to each other compared to the UK. Furthermore, the effect of vaccination timing on the cytokine/chemokine signatures in Malawian infants was examined. Finally, we determined the micronutrient status and degree of inflammation by measuring the concentrations of plasma zinc and CRP in Malawian infants. Our previous findings showing higher Th2 and lower Th1 responses to *M. tb* purified protein derivative (PPD) in Malawian infants compared with UK infants [4,14,15], led us to examine zinc deficiency in Malawian infants based on the reports regarding a Th1 to Th2 cytokine shift in zinc deficiency and the effects of high concentrations of phytate in maize on zinc concentrations [6-8]. In addition, the associations between plasma zinc and CRP concentrations and cytokine responses to *M. tb* PPD were analysed.

## Methods

### Sample and data collection

Samples from studies assessing immune responses following BCG vaccination in infants from Malawi [4,16], The Gambia [17] and data from a parallel study in the UK [4,18] were included in this study. The samples collected from infants at 3 months post BCG vaccination at all study sites were stored at −80°C until assayed to determine cytokine/chemokine signatures and concentrations of plasma zinc and CRP.

Infants were vaccinated with BCG within the first week of life in Malawi (Danish strain 1331, 0.05 mL, intradermal injection; Statens Serum Institut) [4,16] and The Gambia (BCG-Russia, Lot 037G9105, 0.05 mL, intradermal injection; Serum Institute India) [17]. At 3 months post BCG vaccination, the infants were recruited and a diluted whole blood assay (WBA) was performed with *M. tb* PPD (5 μg/mL), PHA (5 μg/mL) and the control (RPMI medium only) to examine the IFN-γ response to mycobacterial antigens [16,17]. For the multiplex bead assay, culture supernatant samples were tested from 30 Malawian infants and 24 Gambian infants (Table 1). For the comparison of cytokine responses to *M. tb* PPD, the cytokine response data from UK infants from a previous study of post BCG immune responses were used [18]. The BCG vaccine strain used in the UK was the same as in Malawi (BCG-Danish 1331) and the median age of UK infants tested at 3 months post BCG vaccination was 7 weeks at the time of vaccination. The same protocols, including the concentration of antigens, were used for WBA in the Malawian, UK and Gambian cohorts [4,17].

**Table 1:** Samples utilised for the assays

		**Malawi**	**The gambia**	**Purpose of collection**
**Culture supernatant**		**30**	**24**	42plex bead assay
Season of birth	Rainy season	16	0	
	Dry season	14	24	
**Plasma**		**63 (57)**		Zn^2+^ & CRP assay
Season of birth	Rainy season	40 (38)		
	Dry season	23 (19)		
BCG given time (week after birth)		1	1	
Mean Age at test (month)		3.2 (2.8-6.5)	3.1 (1.8-3.8)	
Mean body weight at test (kg)		6.3 (4.4-9.2)	6.2 (4.2-7.7)	

Culture supernatants obtained from Malawian (n = 30) and Gambian infants (n = 24) tested at 3 months post BCG vaccination were utilised to measure 42 cytokine/chemokine signatures. In Malawi, the rainy season is from January to May and the dry season is from June to December. In The Gambia, the wet season is from July to December and the dry season is from January to June. For testing CRP and zinc (Zn^2+^) levels in plasma, 63 plasma samples were tested from Malawian infants 3 months post BCG vaccination. Among the plasma samples selected, 6 samples were excluded from CRP testing as these infants were immunized with DPT, Hib and IPV within 7 days prior to blood collection. The number of samples excluding those who had DPT, Hib and IPV immunization within 7 days before the blood collection is indicated in parentheses.

In Malawi, 63 plasma samples from the infants at 3 months post BCG vaccination were tested for plasma zinc and CRP. Among the 403 infants vaccinated within the first week of life [16], those who showed high IFN-γ (> 50 pg/mL) in control cultures (n = 133) and whose mothers had HIV, malaria or helminth infection (n = 165) were excluded. To control confounding factors for the zinc assay, only breast-milk fed infants were included and diurnal variation was avoided by using samples collected in the morning (n = 105). In total, 63 samples from 27 males and 36 females were available (Table 1). As CRP levels are elevated after immunization with vaccines such as DPT (diphtheria, pertussis, tetanus), *Haemophilus influenzae b* (Hib), hepatitis B (HBV) and inactive polio vaccines (IPV) [19], infants who had other immunisations in the 7 days prior to blood collection were excluded from the CRP data analysis.

Ethical approval for the studies was obtained from the London School of Hygiene & Tropical Medicine Ethics Committee, the National Health Sciences Research Committee in Malawi and the Gambian Government/Medical Research Council (MRC) Joint Ethics Committee; permission to export samples was granted by the National Health Sciences Research Committee in Malawi and by the Gambian Government/MRC Joint Ethics Committee in The Gambia.

### Multiplex bead assay

Concentrations of 42 cytokines and chemokines: IL-1α, IL-1ra, IL-1β, IL-2, sIL-2Rα, IL-4, IL-5, IL-6, IL-9, IL-10, IL-12p40, IL-12p70, IL-13, IL-15, IL-17, IFN-α2, IFN-γ, TNF-α, TNF-β, sCD40L, MIP-1α (CCL3), MIP-1β (CCL4), Gro-α (CXCL1), IL-8 (CXCL8), IP-10 (CXCL10), MCP-1 (CCL2), MCP-3 (CCL7), MDC (CCL22), TGF-α, G-CSF, GM-CSF, IL-3, IL-7, Eotaxin, FGF-2, Flt-3 L, Fractalkine (CX3CL1), EGF, VEGF, PDGF-AA, PDGF-AB/BB and RANTES (CCL5) were determined in culture supernatant samples obtained from 30 Malawian and 24 Gambian infants. Multiplex beads were diluted 1 in 2 in bead diluent and the 42plex bead assay was performed according to the manufacturer’s protocol (no. MPXHCYTO60KPMX42; MILLIPLEX®_MAP_ Kit, Millipore, Billerica, MA, USA) as described previously [18]. Multiplex quantification of cytokines in all samples was performed using the Luminex ^R^ 100 System (Luminex, Austin, TX, USA).

The same protocol for the 42 multiplex bead assays was used for testing all samples, although the assays were performed at different times. The antibodies for all 42 cytokines in all the kits used (no. MPXHCYTO60KPMX42; MILLIPLEX®_MAP_ Kit) were derived from the same clones. Samples from both Malawi and The Gambia were transported to LSHTM for testing.

### Zinc colorimetric assay

Zinc concentration was measured in 63 Malawian infant plasma samples by a zinc colorimetric method. A QuantiChrom™ zinc assay kit (BioAssay Systems, Hayward, CA, USA) was used according to the manufacturer’s protocol. Briefly, 100 μL of 5 standards (10.0, 7.5, 5.0, 2.5 and 0 μM of Zn^2+^), 50 μL of diluted plasma samples (1:5) and sample blanks (50 μL sample + 2 μL EDTA) were transferred into the wells of a clear flat-bottom 96 well plate and 200 μL of working solution (200 μl of “reagent A” and 4 μl of each “reagent B” and “reagent C”) was added. After incubation for 30 minutes at room temperature, optical density was measured at 425 nm using a Spectramax M3 plate reader (Molecular Devices, Sunnyvale, CA, USA).

### CRP test

Plasma CRP concentrations were measured by sandwich ELISA using human anti-CRP IgG (THE BINDING SITE, Birmingham, UK; 1:4000), HRP-conjugated anti-human CRP protein (DAKO, Carpinteria, CA, USA; 1:1000) and human CRP standard serum (Behring, Haywards Heath, UK). Plasma samples were diluted 1:200. Substrate solution was prepared by adding 500 μL of 10 mg 3,3′,5,5′-tetramethylbenzidine dissolved in DMSO (Sigma-Aldrich) into the 50 mL of substrate buffer (10 mL of 0.1 M citric acid and 10 mL of 0.2 M Na_2_HPO_4_; pH 4.5 with 10 μL H_2_O_2_; 30%; Sigma-Aldrich). Plates were read at 450 nm with a reference wavelength of 490 nm using a Dynal plate reader (DYNEX Technologies, Worthing, UK).

### Statistical analysis

The Mann Whitney test was used to compare the cytokine/chemokine signatures between two groups from Malawi, The Gambia and the UK. Among Malawian infants, the median concentrations of IFN-γ, CRP and zinc were compared between infants born in the rainy and dry seasons. The association between CRP, zinc and cytokines were examined by calculating the Spearman’s rank correlation coefficient, and the p values obtained from multiple comparisons were adjusted by Bonferroni correction. All of the data obtained from the multiplex bead assay, zinc and CRP tests were analysed with values corrected for background production by subtracting medium alone (negative control) values.

## Results

### Comparison of infant cytokine/chemokine signatures in Malawi, the Gambia and the UK

**Figure 1:**
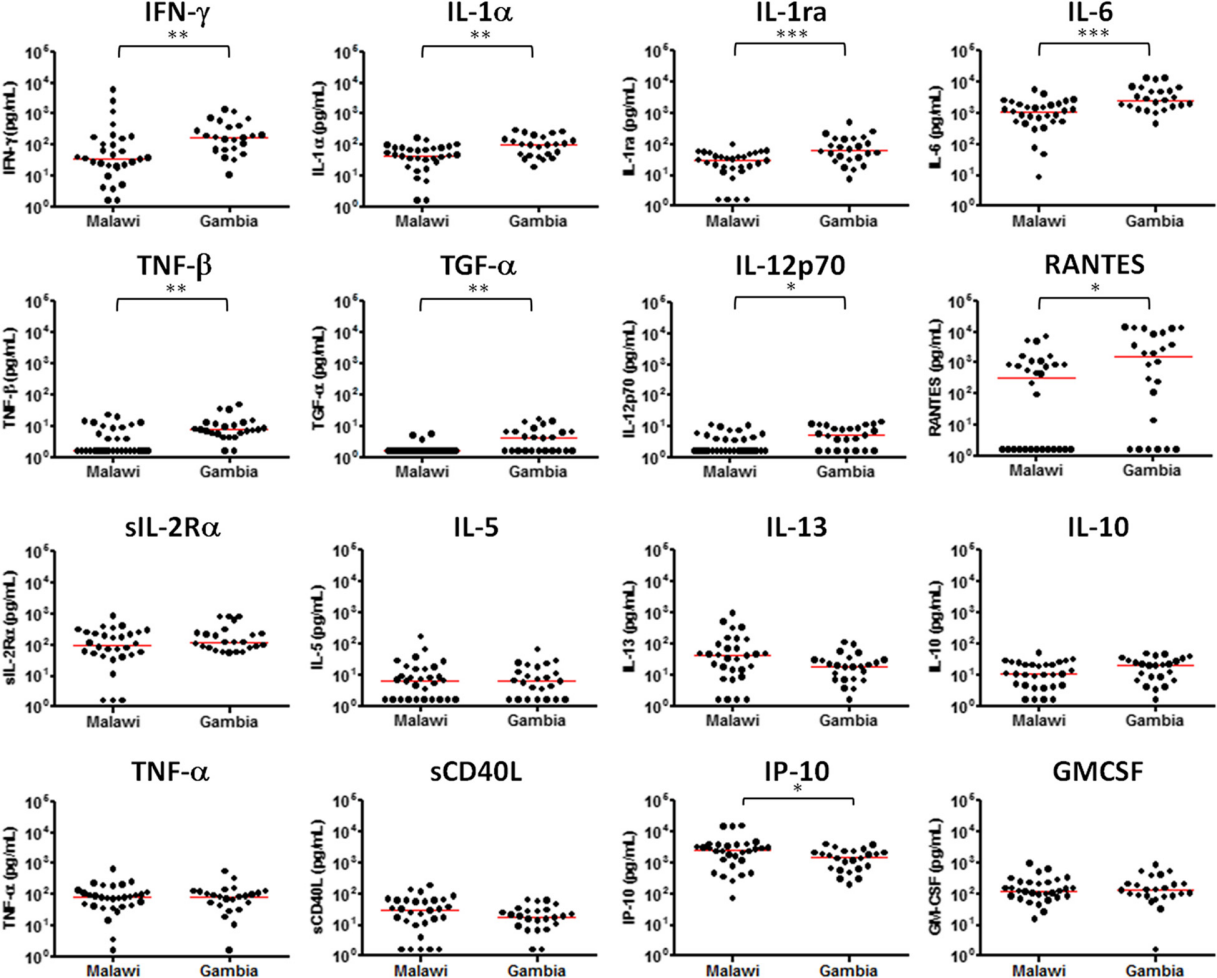
**Cytokine response to *M. tb *PPD in Malawian and Gambian infants vaccinated in the first week of life.** The production of IFN-γ (P = 0.0024), IL-1α (P = 0.0020), IL-1ra (P = 0.0008), IL-6 (0.0002), TNF-β (P = 0.0032), TGF-α (p = 0.0027), IL-12p70 (P = 0.0145), and RANTES (P = 0.0274) was significantly higher in Gambian infants (n = 24) than Malawian infants (n = 30) while most of the other cytokines measured showed similar responses (P > 0.05). The median levels of each cytokine are indicated in red (*P < 0.05, **P < 0.01, ***P < 0.001, Mann Whitney test).

The effect of geographical location on cytokine/chemokine responses to *M. tb* PPD was examined in Malawian and Gambian infants. The Gambian infants vaccinated at birth showed more than twice the median concentration of IFN-γ, IL-1α, IL-1ra, IL-6, TNF-β, TGF-α, IL-12p70 and RANTES in response to *M. tb* PPD compared to Malawian infants vaccinated at birth (P < 0.05) (Figure 1). While there were small differences in median values of most of the other cytokines, these differences did not reach significance (P > 0.05) (Figure 1, Additional file 1: Table S1). The net MCP-1 responses to *M. tb* PPD and PHA were very low in Gambian infants, but this was due to high background levels (Table 2). IL-3, IL-4 and IL-15 were not detected in supernatants from either Malawian or Gambian infants. When compared to results from UK infants previously tested at 3 months post BCG vaccination, the Gambian infants showed significantly lower median IFN-γ, IL-1α, IFN-α2, IL-17, IL-12p40, sCD40L, IP-10, IL-8, FGF-2 and IL-7 responses (P < 0.001) (Table 2, Additional file 1: Table S1).

**Table 2:** **The median responses of cytokines to *M. tb *** **PPD by timing of vaccination and population**

**Analyte (pg/mL)**	**Malawi**	**Malawi**	**The Gambia**	**UK**	**Malawi**	
	**BCG between 3–11 weeks**^**a**^	**BCG at 1 week**	**BCG at 1 week**	**BCG between 3–13 weeks**^**a**^	**Early vs late BCG vaccination**	
	**n = 40**	**n = 30**	**n = 24**	**n = 28**	**Fold difference**^**b**^	**P value**^**c**^
**Pro-inflammatory**						
IFN-γ	76	36.8	175.5	902	2.1	0.26
IL-2	1.6	4.2	1.6	10	0.4	<0.001
sIL-2R	1400	96.8	117.7	227	14.5	<0.001
IL-1α	1173	45.5	102.3	399	25.8	<0.001
IL-1β	17	22.1	36.7	27	0.8	0.61
IL-1ra	222	29.1	62.1	116	7.6	<0.001
IL-6	954	1149.1	2654.1	1881	0.8	0.069
TNF-α	139	81.3	87.5	111	1.7	0.030
TNF-β	4	1.6	7.7	14	2.5	0.19
IFN-α2	108	1.6	3.6	22	67.5	<0.001
**Th2**						
IL-4	4	1.6	1.6	1.6	2.5	<0.001
IL-5	75	6.8	6.4	4	11.0	<0.001
IL-13	1434	41.6	19.4	47	34.5	<0.001
**Th9**						
IL-9	10	1.6	1.6	1.6	6.3	<0.001
**Th17**						
IL-17	60	7	5.4	26	8.6	<0.001
**T cell regulation**						
IL-10	95	10.6	20	23	9.0	<0.001
**T cell activation**						
IL-12p40	27.5	8.4	6.6	63	3.3	<0.001
IL-12p70	6	1.6	5.5	1.6	3.8	<0.001
**Costimulation**						
sCD40L	186	30.8	18.6	153	6.0	<0.001
**Chemokines**						
IP-10	3758	2517.2	1530.1	12798	1.5	0.006
MIP-1α	64	130.2	93	623	0.5	0.31
MIP-1β	709.5	348.3	300.5	961	2.0	0.019
MCP-1	15000	6381.3	1.6	9816	2.4	0.015
MCP-3	3065	2003	3242	860	1.5	0.004
MDC	2216	499.1	969.8	1415	4.4	<0.001
Gro	2725	2094	1646	936	1.3	0.11
RANTES	1093	330	1543	611	3.3	0.012
Eotaxin	33.5	46.1	33.4	29	0.7	0.001
Fractalkine	253.5	353.9	222.3	272	0.7	0.032
IL-8	14998	14549	8287	15892	1.0	<0.001
**Growth factors**						
GCS-F	19.5	31	90.9	13	0.6	0.99
GM-CSF	717	117.5	127.6	376	6.1	<0.001
IL-3	49	1.6	1.6	1.6	30.6	<0.001
TGF-α	4.5	1.6	4.1	1.6	2.8	<0.001
FGF-2	111	21.1	35.6	99	5.3	<0.001
Flt-3 L	30.5	4.3	8	17	7.1	<0.001
IL-7	84.5	1.6	1.6	89	52.8	<0.001
EGF	14.5	14.5	11.1	16	1.0	0.99
VEGF	88.5	209.3	233.5	106	0.4	<0.001
PDGF-AA	506.5	179.2	112.4	108	2.8	<0.001
PDGF-AB/BB	381	305.2	801.5	106	1.2	0.92

The median values of most cytokines and chemokines were higher in the infants vaccinated late compared with the infants vaccinated in the first week after birth in Malawi. In contrast, infants vaccinated within 7 days of birth in Malawi and The Gambia showed comparable median responses to PPD in many of the cytokines, compared with the Malawian infants who were vaccinated late. IL-15 was not included due to the undetectable levels in all of the infant samples.

^a^The data from the Malawian and UK infants vaccinated between 3–13 weeks of life were obtained from a previous study [18]. The median ages of late vaccinated Malawian and UK infants are 5 and 7 weeks, respectively.

^b^The fold differences of each cytokine response to *M. tb* PPD were calculated by dividing the median values of the Malawian infants with delayed vaccination (between 3–11 weeks of life) by those of early vaccinated infants (within 1 week of life).

^c^P values for the median differences of each cytokine response to *M. tb* PPD between the two groups (Mann Whitney test).

### The effect of season of birth on cytokine/chemokine signatures

**Figure 2:**
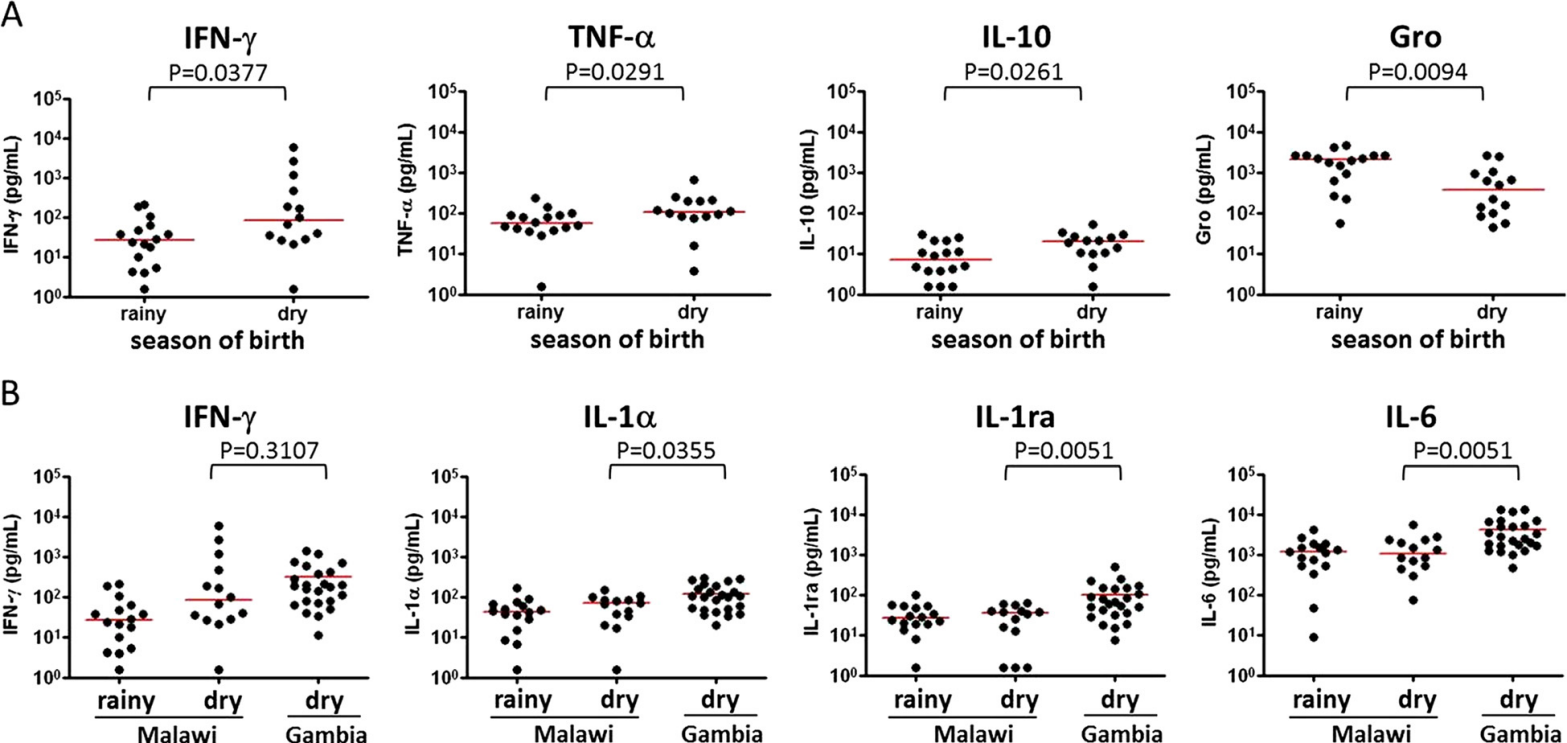
**Effect of season of birth on cytokine/chemokine signatures. A**. Comparison of cytokine/chemokine signatures between dry and rainy seasons of birth in 30 Malawian infants. Among the 42 cytokines and chemokines measured, 3 cytokines including IFN-γ, TNF-α and IL-10 were higher in the infants who were born in the dry season compared with those born in the rainy season. Conversely, Gro production was significantly higher in infants born in the rainy season than in those with dry season of birth. **B**. Further analysis considering season of birth between the Malawian (n = 14) and Gambian infants (n = 24) born in the dry season demonstrated that only IL-1α, IL-1ra and IL-6 responses to *M. tb* PPD were significantly higher in the Gambian infants (P < 0.05, Mann Whitney test). Other cytokines including IFN-γ responses to *M. tb* PPD in Malawian and Gambian infants who were born in the dry season were not significantly different (P > 0.05, Mann Whitney test). The median responses to *M. tb* PPD are indicated in red.

To investigate the seasonal variation in IFN-γ responses to *M. tb* PPD observed previously [4], a large panel of 42 cytokines and chemokines were measured in Malawian infants vaccinated at birth to examine the difference in immune responses in more detail. IFN-γ, TNF-α and IL-10 were significantly higher in the infants born in the dry season (P < 0.05) while Gro (CXCL1) was higher in the rainy season (P < 0.01) (Figure 2A). The levels of 38 other analytes including 17 cytokines, 10 chemokines and 11 growth factors were not statistically different between the two seasons of birth. The samples obtained from the Gambian infants were all from infants born in the dry season, and their cytokine/chemokine signatures were compared with 14 Malawian infants who were also born in the dry season. This revealed that only three cytokines, namely IL-1α, IL-1ra and IL-6, were higher in the 24 Gambian infants compared with the 14 Malawian infants also born in the dry season (P < 0.05) (Figure 2B), though with smaller numbers for this comparison the power to show differences was low. The median IFN-γ response to *M. tb* PPD was not significantly different in Malawian and Gambian infants born in the dry season (P = 0.31) (Figure 2B). The median IFN-γ response in Malawian infants was enhanced from 36.8 to 81.1 pg/mL when the 16 infants born in the rainy season were excluded compared to 177.5 pg/ml for the Gambian infants also born in the dry season.

### Cytokine/chemokine signatures in Malawian infants according to time of BCG vaccination

**Figure 3:**
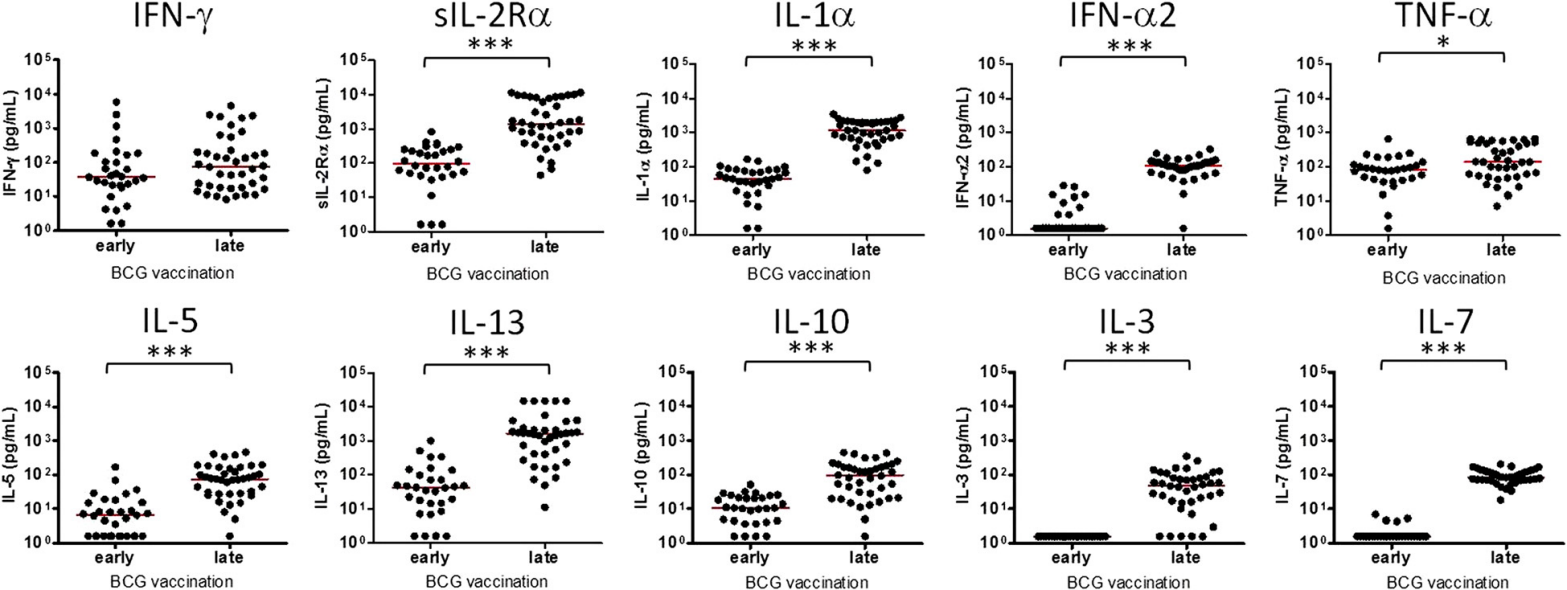
**Production of cytokines and growth factors in early vaccinated versus late vaccinated infants.** The levels of 6 cytokines (sIL-2Rα, IL-1α, IFN-α2, IL-5, IL-13, IL-10) and two growth factors (IL-3 and IL-7) were significantly higher with more than 9-fold difference in the infants who were given BCG between 3 and 11 weeks of life (vaccinated late) compared with those vaccinated within one week of birth (vaccinated early). The concentrations of IFN-γ and TNF-α were also higher in late vaccinated infants but the fold differences in median responses were moderate compared with the above 6 cytokines and 2 growth factors. The median levels of each cytokine are indicated in red. The data from late vaccinated Malawian infants were obtained from the previous study [18] (*P < 0.05, ***P < 0.001, Mann Whitney test).

To determine the effect of age at vaccination on immune responses to *M. tb* PPD, the cytokine/chemokine signatures were compared between Malawian infants with early vaccination (within 1 week of birth) and a group of Malawian infants from the same cohort, studied previously, who had received BCG vaccination late (between 3 and 11 weeks of life) [18]. IFN-γ production was not significantly different between early and late vaccinated infants (Figure 3) while the levels of 25 other cytokine/chemokine responses were more than two times higher in the infants vaccinated late compared with those who had BCG within 1 week of birth (Table 2). Indeed, the infants vaccinated late showed more than a 5 fold higher production of 15 cytokines including several growth factors (P < 0.001) (Table 2). The median concentrations of three pro-inflammatory cytokines (sIL-2Rα, IL-1α, IFN-α2), a regulatory T cell cytokine (IL-10), two Th2 cytokines (IL-5, IL-13) and two growth factors (IL-3, IL-7) were more than 9 times higher in infants vaccinated between 3 and 11 weeks of life compared with those vaccinated early within one week of birth (Table 2, Figure 3).

### Plasma concentrations of zinc and CRP in BCG vaccinated Malawian infants

**Figure 4:**
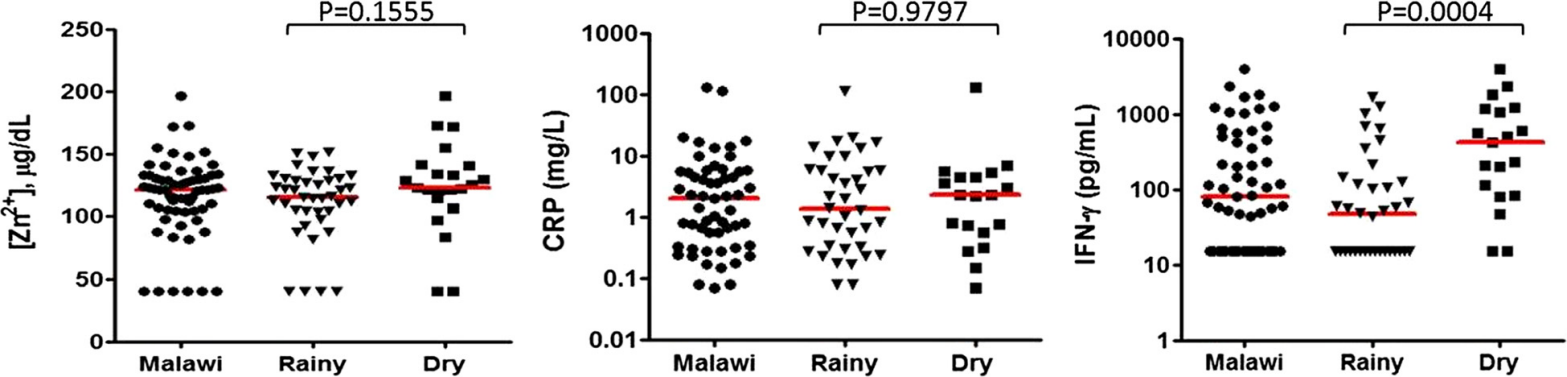
**Concentrations of plasma zinc (Zn**^**2+**^**), CRP and IFN-γ in Malawian infants.** Zinc deficiency was not detected in the majority of the Malawian infants although 6 of 63 infants showed zinc deficiency (< 65 μg/dL). Levels of CRP greater than 10 mg/L were detected in 8 Malawian infants. The median IFN-γ responses to *M. tb* PPD were different between early vaccinated infants with rainy (n = 40) and dry season (n = 23) of birth (P < 0.001, Mann Whitney test) while the plasma zinc and CRP concentrations were unaffected by season of birth (P > 0.05, Mann Whitney test). The median levels of zinc, CRP and IFN-γ are indicated in red.

Plasma concentrations of zinc and CRP were measured to determine zinc deficiency and the degree of inflammation in Malawian infants, and to investigate possible associations with season of birth and cytokine/chemokine signatures. Zinc deficiency (< 65 μg/dL) was shown in 9.5% (6/63) of Malawian children. Eight of 57 Malawian infants showed plasma CRP levels greater than 10 mg/L, while 2 infants had CRP levels greater than 100 mg/L (Figure 4). The IFN-γ response to *M. tb* PPD was higher in infants born in the dry season (P < 0.001) while the plasma concentrations of zinc and CRP were unaffected by season of birth in Malawi (P > 0.05) (Figure 4) although 7 of the 8 Malawian infants with CRP > 10 mg/L were born in the rainy season. Spearman’s rank correlation showed no evidence that the plasma zinc and CRP concentrations were associated with the IFN-γ responses to *M. tb* PPD (r = 0.04, P > 0.05 for CRP; r = 0.20, P > 0.05 for zinc; data not shown). Looking at the correlation between plasma CRP concentration and the cytokine/chemokine signatures observed in 17 Malawian infants who had samples for testing of both culture supernatant and plasma, there was no evidence that any cytokine or chemokine induced by *M. tb* PPD was associated with plasma CRP concentration (P > 0.05; data not shown).

## Discussion

This study focused on the evaluation of infant immune responses at 3 months post BCG vaccination, investigating potential factors that may affect cytokine/chemokine signatures in infants from different countries and vaccinated at different ages. It has been reported that variation in resistance to TB and efficacy of BCG may be influenced by latitude dependent factors such as climate, exposure to nontuberculous mycobacteria and genetics [3]. In our comparisons of the immunogenicity of BCG the geographical location and environment of Malawi and The Gambia are much closer to each other than they are to the UK and this corresponds with the similarity of the cytokine/chemokine signatures observed. In addition, the difference in cytokine/chemokine signatures between Malawian and Gambian infants was partly due to the season of birth; the difference in median IFN-γ response in Gambian compared to Malawian infants born in the dry season was not statistically significant (P = 0.31). One potential confounder may be the strain of BCG used for vaccination. It is still unknown how the immune response can be affected by different vaccine strains as it may depend on stimulating antigen, population and assay protocol. In this study, the infants were not immunised with the same BCG vaccine strains; Malawian and UK infants were immunized with the Danish strain, SSI 1331 while Gambian infants received BCG-Russia. These two strains are genetically different and have been shown to induce a different immune response to crude CFP and Ag85 antigens in children in Uganda [20,21]. Immunization with BCG-Denmark or BCG-Japan induced higher frequencies of mycobacterial-specific polyfunctional and cytotoxic T cells compared with BCG-Russia in Australia [22]. However, statistical differences were shown only when BCG was used for *in vitro* stimulation, and not with PPD or MTB antigens [22]. In addition, there were no significant differences in cytokine responses to BCG and PPD measured in culture supernatant between infants immunized with BCG-Denmark or BCG Russia [22]. In our study, production of only 3 of 42 cytokines (IL-1, IL-1ra and IL-6) was significantly different in the infants from Malawi and the Gambia after adjusting for season of birth. Therefore, the differences in cytokine responses to *M. tb* PPD between Malawi and The Gambia may be due to season of birth rather than to the BCG vaccine strains.

The cytokine/chemokine signatures observed were influenced by the timing of BCG vaccination. In Malawi, when compared with infants vaccinated early, the infants vaccinated later (a median age of 5 weeks) showed higher responses to *M. tb* PPD for most cytokines and chemokines measured but not for IFN-γ. A similar finding of varying BCG immunogenicity by age at vaccination was reported in South African infants, showing that enhanced BCG-specific T cell responses were observed in infants when vaccination was delayed from birth to 10 weeks [23]. However, two studies in The Gambia [24] and Uganda [25] showed contradicting results; the magnitude of the IFN-γ response was not significantly altered by the timing of vaccination when comparing infants vaccinated at birth or 2 months in The Gambia [24], whereas greater frequencies of BCG-specific IFN-γ positive CD4^+^ T cells were detected in the group vaccinated at birth compared with those vaccinated at 6 weeks of age in Uganda [25].

In this study, there was no evidence of a difference in median IFN-γ production between Malawian infants vaccinated early compared with those vaccinated late, but there was a trend of higher responses in those vaccinated late. The Malawian infants vaccinated late showed higher median (p < 0.001) Th2 (IL-4, IL-5 and IL-13) recall responses to *M. tb* PPD than infants vaccinated at birth. This observation suggests that different immune signatures develop according to timing of vaccination which might be an important factor in relation to the induction of protective immune responses following vaccination. The samples obtained from early and late BCG vaccinated infants in Malawi were derived from the same large cohort study [4]. A total of 615 Infants were vaccinated with BCG between 2002 and 2004, and blood samples were obtained from 590 infants at 3 months post BCG vaccination between 2003 and 2005 [4]. Blood samples were obtained from 590 of these infants at 3 months post BCG vaccination. Infants were vaccinated early or late throughout the whole recruiting period. This was as a result of whether the infants were born in hospital and vaccinated at birth or born elsewhere and brought to the clinic later for vaccination. There was no difference in the vaccination they received. Thus no other confounding factors that might be associated with delayed vaccination have been identified. The results of this study are also consistent with evidence that newborns can develop Th1 responses following BCG vaccination [24,25] unlike the Th2 bias in mice [26].

In terms of the proportion of infants who showed greater than 10 mg/L of CRP in their plasma, the majority (7/8) were born in the rainy season. In the whole infant cohort no infants showed helminth infections at 3–6 months of age. No infants were bled if unwell or with symptoms of malaria or other infection. We excluded the infants whose mothers had HIV, malaria, or helminth infections but we did not test for other diseases which could induce inflammation in the infants and might affect the CRP concentration in the infants and their mothers. Therefore higher plasma CRP concentration, reflecting level of inflammation, most likely indicates a higher burden of infections, but there was no association between the IFN-γ responses to *M. tb* PPD and CRP concentration (data not shown). Most of the Malawian infants had a normal range of plasma zinc concentrations and none of the cytokines tested were associated with the concentration of zinc (data not shown), indicating that zinc status might not be a major factor affecting the differences in infant immune responses in Malawi, at least when measured at 3 months post BCG vaccination.

To examine if cytokine activity had been lost over time in storage, we tested culture supernatant samples which had been collected from 4 infants at 3 years post BCG vaccination and stored in the same place as the archived samples we used for this study, since 2006. There was no significant difference in IFN-γ concentrations of 19 culture supernatant samples from each of 4 subjects when measured in 2006 and again in 2010, and a strong correlation was found between IFN-γ values obtained in 2006 and 2010 (r = 0.9808, P < 0.0001) [27].

Differences in the infant immune responses to *M. tb* PPD following BCG vaccination are documented at different latitudes and in different populations. The extent to which these influence BCG efficacy remains to be evaluated alongside studies looking for correlates of protection against TB [28]. Season of birth and timing of vaccination have been shown here to be critical factors affecting the cytokine/chemokine signatures induced by BCG vaccination in Malawian infants. In addition, this study indicates the potential importance of the balance between type 1 and 2 cytokines, or between pro- and anti-inflammatory cytokines rather than the absolute quantities of a particular cytokine. Although we were limited by the number of samples tested, the results of this study suggest the need for additional biomarkers to be measured in conjunction with IFN-γ to assess the immunogenicity of BCG, and that potential factors affecting immune responses following vaccination such as season of birth should be taken into account for future trials of new TB vaccines.

## Conclusions

In conclusion, variation in geographical location, season of birth, and age at vaccination may alter the cytokine/chemokine signature to *M. tb* PPD, in infants at 3 months post BCG vaccination, while the concentration of plasma zinc or CRP did not correlate with the cytokine/chemokine signature to *M. tb* PPD. We therefore recommend that the season of birth and timing of priming vaccines, as well as geographical setting, should be considered in the design of clinical trials of new TB vaccines.

## Abbreviations

CRP: C-reactive protein; DPT: Diphtheria pertussis tetanus; HBV: Hepatitis B vaccine; Hib: *Haemophilus influenzae* b; IPV: Inactive polio vaccine; KPS: Karonga prevention study; M. tb: *Mycobacterium tuberculosis*; PPD: Purified protein derivative; TB: Tuberculosis.

## Competing interests

The authors declare that they have no competing interests.

## Author’s contributions

YGH, PGS, HMD conceived the study. ABS, HM, JRF, KLF, SB, MOO, ACC participated in enrolment of study subjects in Malawi and The Gambia. YGH, MKL carried out experiments. YGH, SF performed statistical analysis. JR, SGS, HMD contributed reagents for the multiplex bead assay and CRP test. YGH drafted the manuscript and PGS, HMD helped to draft the manuscript. All authors read and approved the final manuscript.

## Pre-publication history

The pre-publication history for this paper can be accessed here:

http://www.biomedcentral.com/1471-2334/14/184/prepub

## Supplementary Material

Additional file 1: Table S1 The fold differences of each cytokine response to *M. tb* PPD between Gambian and Malawian infants, Gambian and UK infants. The cytokine responses to *M. tb* PPD were compared among Malawian and Gambian infants who were vaccinated at 1 week after birth, and UK infants vaccinated between 3-13 weeks of life [18]. ^d,f^ The fold differences of each cytokine response to *M. tb* PPD were calculated by dividing the median values of Gambian infants by Malawian infants with early vaccination and by dividing the median values of UK infants by those of Gambian infants, respectively. ^e^P values for the median differences of each cytokine response to *M. tb* PPD between the two groups (Mann Whitney test). Click here for file
