# Supplementary material for: Factors affecting immunogenicity of BCG in infants, a study in Malawi, The Gambia and the UK
Source: BMC Infect Dis. 2014 Apr 5;14:184. doi: 10.1186/1471-2334-14-184 (PMC4101864; doi:10.1186/1471-2334-14-184)
Supplement: Additional file 1: Table S1 — The fold differences of each cytokine response to M. tb PPD between Gambian and Malawian infants, Gambian and UK infants. The cytokine responses to M. tb PPD were compared among Malawian and Gambian infants who were vaccinated at 1 week after birth, and UK infants vaccinated between 3-13 weeks of life [18]. d,f The fold differences of each cytokine response to M. tb PPD were calculated by dividing the median values of Gambian infants by Malawian infants with early vaccination and by dividing the median values of UK infants by those of Gambian infants, respectively. eP values for the median differences of each cytokine response to M. tb PPD between the two groups (Mann Whitney test). [file 1471-2334-14-184-S1.pdf]

Analyte	The Gambia / Malawi		UK / The Gambia	
	Fold difference[d]	P value[e]	Fold difference[f]	P value[e]
**Pro-inflammatory**				
IFN-γ	4.8	0.002	5.1	<0.001
IL-2	0.4	0.046	6.3	0.001
sIL-2R	1.2	0.22	1.9	0.15
IL-1α	2.2	0.002	3.9	<0.001
IL-1β	1.7	0.11	0.7	0.25
IL-1ra	2.1	0.001	1.9	0.022
IL-6	2.3	<0.001	0.7	0.043
TNF-α	1.1	0.84	1.3	0.15
TNF-β	4.8	0.003	1.8	0.18
IFN-α2	2.3	0.23	6.1	<0.001
**Th2**				
IL-4	1.0	0.28	1.0	0.033
IL-5	0.9	0.98	0.6	0.96
IL-13	0.5	0.069	2.4	0.018
**Th9**				
IL-9	1.0	0.024	1.0	0.56
**Th17**				
IL-17	0.8	0.33	4.8	<0.001
**T cell regulation**				
IL-10	1.9	0.14	1.2	0.055
**T cell activation**				
IL-12p40	0.8	0.89	9.5	<0.001
IL-12p70	3.4	0.015	0.3	0.091
**Costimulation**				
sCD40L	0.6	0.18	8.2	<0.001
**Chemokines**				
IP-10	0.6	0.038	8.4	<0.001
MIP-1α	0.7	0.98	6.7	0.017

MIP-1β	0.9	0.70	3.2	0.005
MCP-1	0.0	<0.001	6135.0	<0.001
MCP-3	1.6	0.096	0.3	0.002
MDC	1.9	0.036	1.5	0.69
Gro	0.8	0.63	0.6	0.49
RANTES	4.7	0.027	0.4	0.64
Eotaxin	0.7	0.032	0.9	0.82
Fractalkine	0.6	0.004	1.2	0.78
IL-8	0.6	<0.001	1.9	<0.001
**Growth factors**				
GCS-F	2.9	0.11	0.1	<0.001
GM-CSF	1.1	0.94	2.9	0.001
IL-3	1.0	0.39	1.0	0.20
TGF-α	2.6	0.003	0.4	0.010
FGF-2	1.7	0.17	2.8	<0.001
Flt-3L	1.9	0.16	2.1	0.040
IL-7	1.0	0.021	55.6	<0.001
EGF	0.8	0.81	1.4	0.89
VEGF	1.1	0.67	0.5	0.001
PDGF-AA	0.6	0.58	1.0	0.99
PDGF-AB/BB	2.6	0.012	0.1	<0.001

Supplementary Table 1. The fold differences of each cytokine response to *M. tb* PPD between Gambian and Malawian infants, Gambian and UK infants. The cytokine responses to *M. tb* PPD were compared among Malawian and Gambian infants who were vaccinated at 1 week after birth, and UK infants vaccinated between 3-13 weeks of life [18].

[d,f] The fold differences of each cytokine response to *M. tb* PPD were calculated by dividing the median values of Gambian infants by Malawian infants with early vaccination and by dividing the median values of UK infants by those of Gambian infants, respectively.

[e] P values for the median differences of each cytokine response to *M. tb* PPD between the two groups (Mann Whitney test).
